# Proteomics at our fingertips: Coupling remote collection devices with NULISA multiplex panels

**DOI:** 10.1002/alz70856_102262

**Published:** 2025-12-25

**Authors:** Rachael E. Wilson, Ramiro Eduardo Rea Reyes, Aaron Fredricks, Sara Rusch, Monica VandenLangenberg, Cindy Jensen, Martie Marshall, Elysse Keske, Beckie Jeffers, Haley Weninger, Hanna Huber, Laia Montoliu‐Gaya, Nicholas J. Ashton, Sterling C Johnson, Henrik Zetterberg, Alamar TAP team

**Affiliations:** ^1^ Wisconsin Alzheimer's Institute, University of Wisconsin School of Medicine and Public Health, Madison, WI, USA; ^2^ Department of Medicine, University of Wisconsin‐Madison School of Medicine and Public Health, Madison, WI, USA; ^3^ University of Wisconsin‐Madison, Madison, WI, USA; ^4^ UW Health, Madison, WI, USA; ^5^ Wisconsin Alzheimer's Disease Research Center, University of Wisconsin School of Medicine and Public Health, Madison, WI, USA; ^6^ University of Gothenburg, Mölndal, Sweden; ^7^ University of Gothenburg, Gothenburg, Sweden; ^8^ Institute of Neuroscience and Physiology, Department of Psychiatry and Neurochemistry, The Sahlgrenska Academy at University of Gothenburg, Mölndal, Sweden; ^9^ King's College London, Institute of Psychiatry, Psychology & Neuroscience, Maurice Wohl Clinical Neuroscience Institute, London, United Kingdom; ^10^ Banner Alzheimer's Institute, Phoenix, AZ, USA; ^11^ Department of Psychiatry and Neurochemistry, Institute of Neuroscience & Physiology, the Sahlgrenska Academy at the University of Gothenburg, Mölndal, Sweden; ^12^ Alzheimer's Disease Research Center, University of Wisconsin‐Madison, Madison, WI, USA; ^13^ Wisconsin Alzheimer's Institute, University of Wisconsin School of Medicine and Public Health, Madison, WI, USA; ^14^ Hong Kong Center for Neurodegenerative Diseases, Hong Kong, Science Park, China; ^15^ Wisconsin Alzheimer's Disease Research Center, University of Wisconsin‐Madison, School of Medicine and Public Health, Madison, WI, USA; ^16^ Institute of Neuroscience and Physiology, Sahlgrenska Academy at the University of Gothenburg, Gothenburg, Sweden; ^17^ University College London, London, United Kingdom, London, United Kingdom; ^18^ Department of Psychiatry and Neurochemistry, University of Gothenburg, Gothenburg, Sweden

## Abstract

**Background:**

The Alzheimer's Association's multi‐tiered approach to Alzheimer's disease (AD) staging (Jack, C., et al. 2024) via Core 1 and Core 2 biomarkers reflects the interplay between amyloid, tau, neurodegeneration, inflammation, and co‐occurring pathologies and subsequent effects on symptom manifestation and progression. A single biomarker will probably not be sufficient for characterizing these complexities. The NULISAseq CNS disease panel allows simultaneous detection of 100+ biomarkers related to central nervous system (CNS) dysfunction across a broad dynamic range. Low sample volume requirements (25uL) are amenable to capillary blood analyses via remote collection devices, a scalable approach for obtaining CNS protein profiles from those that are unable to undergo traditional biomarker evaluation.

**Method:**

A stepwise approach was used to explore the effects of buffer composition, volume, and incubation time on the percent recovery (%R) of target analytes collected via Telimmune plasma separator cards relative to analyte concentrations in paired plasma. After selecting the optimum conditions, paired venous plasma samples and capillary dried plasma spots (DPS) from *N* = 40 Wisconsin Registry for Alzheimer's Prevention and the Wisconsin Alzheimer's Disease Center participants (*N* = 36 cognitively unimpaired, *N* = 4 impaired) were analyzed using the NULISAseq CNS disease panel. Analyte recovery, detectability, and Spearman correlations were compared across sample types.

**Result:**

The highest DPS detectability (80%) was achieved using 80uL of Alamar sample diluent spiked with a cocktail of peptidases and phosphatases. Decreasing buffer volume resulted in increasing detectability (R^2^=1.00) down to at least 80uL. Incubation time did not have an obvious effect on detectability. In participant DPS samples, %R(SD) relative to plasma ranged from 77(4)% to 92(5)% for pTau species. Sixty‐two of 127 analytes were significantly correlated in DPS and plasma.

**Conclusion:**

Here, we report optimization and evaluation of capillary DPS samples with a targeted proteomic panel. Analyte recovery and correlations in DPS compared to plasma varied widely depending on the analyte. This is to be expected given the diversity of targets across the panel. Ongoing work includes evaluation in an amyloid‐enriched cohort, additional optimization for detecting AD‐related markers, and head‐to‐head evaluation of other remote sampling devices.

